# Spindle Cell Sarcoma of the Abdominal Aorta Following Endovascular Aneurysm Repair in a Kidney Transplant Recipient

**DOI:** 10.1155/crit/8333765

**Published:** 2026-06-05

**Authors:** Kota Kakeshita, Teruhiko Imamura, Hidenori Yamazaki, Kanetsugu Nagao, Shigeyuki Yamashita, Shinya Kajiura, Naotaka Nishiyama, Noriko Okuno, Tsutomu Koike, Hiroshi Kitamura, Koichiro Kinugawa

**Affiliations:** ^1^ The Second Department of Internal Medicine, University of Toyama, Toyama, Japan, u-toyama.ac.jp; ^2^ The First Department of Surgery, University of Toyama, Toyama, Japan, u-toyama.ac.jp; ^3^ Department of Medical Oncology and Palliative Medicine, University of Toyama, Toyama, Japan, u-toyama.ac.jp; ^4^ Department of Urology, University of Toyama, Toyama, Japan, u-toyama.ac.jp; ^5^ Department of Pathology, University of Toyama, Toyama, Japan, u-toyama.ac.jp

**Keywords:** abdominal aortic aneurysm, EVAR, immunosuppression, infected aneurysm, posttransplant malignancy, soft tissue sarcoma

## Abstract

A kidney transplant recipient developed spindle cell sarcoma within an abdominal aortic aneurysm following prior endovascular aneurysm repair (EVAR). The patient had undergone EVAR for abdominal aortic aneurysm before successful living‐donor kidney transplantation and maintained stable graft function under immunosuppressive therapy. Two years after transplantation, he presented with back pain, elevated inflammatory marker, and aneurysm enlargement, initially suspected as an infected aneurysm. Despite antibiotic therapy, the lesion progressed. Positron emission tomography demonstrated increased fluorodeoxyglucose uptake, and computed tomography‐guided biopsy revealed undifferentiated spindle cell sarcoma with unclear lineage differentiation. Surgical resection was not feasible, and systemic chemotherapy was initiated; however, disease progression continued, and the patient died 7 months after admission. Aortic sarcoma following EVAR is rare and may mimic infection or inflammatory complications. Clinicians should consider malignant etiologies when transplant recipients with prior EVAR present with atypical aneurysm enlargement or persistent inflammatory findings, and early tissue diagnosis may be necessary.

## 1. Introduction

Chronic kidney disease is an independent risk factor for aortic aneurysm and aortic disease‐related mortality [1, 2]. With advances in transplantation medicine, the number of patients with chronic renal failure undergoing endovascular aneurysm repair (EVAR) for concomitant aortic aneurysms before and after kidney transplantation has being increasing [3]. Complications after EVAR include endoleak, endotension, graft migration, graft occlusion, and graft infection [4].

Kidney transplant recipients require immunosuppression, which increases their risk of infection and malignant neoplasms. Overall, kidney transplant recipients have an approximately two to three‐fold higher risk of malignancy compared with the general population. In particular, the increased risk is driven by urinary tract cancers, nonmelanoma skin cancers, and oncogenic viral‐mediated malignancies such as posttransplant lymphoproliferative disorder and Kaposi sarcoma [5]. Among malignancies occurring after kidney transplant, the frequency of sarcomas is estimated to be 7.4%, of which Kaposi sarcoma accounts for 5.7% and other sarcomas for 1.7% [6]. Spindle cell sarcoma accounts for approximately half of all soft tissue sarcomas occurring in adults, and is a descriptive term for sarcomas exhibiting a spindle‐shaped cell morphology [7].

Here, we report a case of a patient who underwent EVAR for an abdominal aortic aneurysm prior to kidney transplantation and subsequently developed progressive aneurysm enlargement accompanied by inflammatory findings 2 years after transplantation, which was ultimately diagnosed as undifferentiated spindle cell sarcoma of unknown lineage differentiation.

## 2. Case Presentation

### 2.1. Before Kidney Transplantation

A male patient with hypertension and proteinuria since around age 40 was diagnosed with chronic renal failure secondary to chronic glomerulonephritis, although a kidney biopsy was not performed, and initiated peritoneal dialysis at age 51.

At age 53, he was incidentally diagnosed with an abdominal aortic aneurysm. Because the maximum transverse diameter reached 50 mm, surgical intervention was indicated. The patient wished to continue peritoneal dialysis and return to social activities as soon as possible; therefore, EVAR using the Gore Excluder (W. L. Gore & Associates G.K., Tokyo, Japan) was performed.

Following EVAR, the postoperative course was uneventful, and peritoneal dialysis was continued. At age 57, he subsequently underwent ABO‐compatible living‐donor kidney transplantation, with his wife serving as the donor. The induction immunosuppressive therapy regimen was as follows: basiliximab (20 mg each just before and 4 days after the transplant), methylprednisolone (gradually tapering from 500 mg to 4 mg), mycophenolate mofetil (2000 mg per day), and tacrolimus (10 mg per day, and the trough level was 8.50 ng/mL).

### 2.2. On Admission

The posttransplant course had been uneventful under maintenance immunosuppressive therapy with methylprednisolone, mycophenolate mofetil, and tacrolimus. No enlargement of the abdominal aortic aneurysm was observed just after the kidney transplantation (Figure 1A).

**Figure 1:**
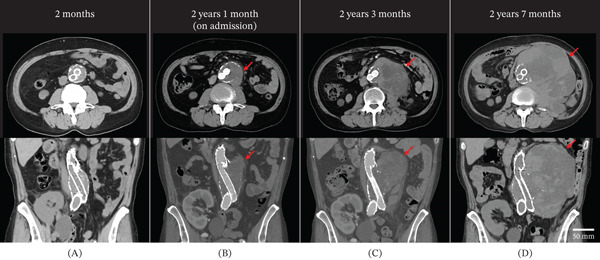
Computed tomography findings after the kidney transplantation (axial view [above] and coronal view [below]). (A) 2 months after the kidney transplantation. The abdominal aortic aneurysm was repaired by implanting stent grafts, and no enlargement of the aneurysm was observed. (B) 2 years and 1 month after the kidney transplantation. An enlargement of the aortic aneurysm and a partial rupture of the aneurysm wall were observed (red arrow) by contrast‐enhanced computed tomography. (C) 2 years and 3 months after the kidney transplantation. The aneurysm had further enlarged and formed a mass (red arrow) by contrast‐enhanced computed tomography. (D) 2 years and 7 months after the kidney transplantation. The tumor grew even larger (red arrow).

However, at age 59, 2 years and 1 month after kidney transplantation, he developed left lower back pain and increased serum inflammatory markers. Imaging studies revealed an enlarged abdominal aortic aneurysm (Figure 1B), and he was hospitalized with a suspected infected aortic aneurysm.

The patient′s physical measurements were as follows: height, 171 cm; weight, 75.2 kg; body mass index, 25.7 kg/m^2^; blood pressure, 134/90 mmHg; pulse rate, 88 beats per minute; and body temperature, 36.7°C. No abdominal bruits were detected upon auscultation. Laboratory findings are summarized in Table 1, including an elevated serum C‐reactive protein level of 8.15 mg/dL.

**Table 1:** Laboratory data on admission.

Laboratory test	Result
Urinalysis	
Specific gravity	1.003
Protein	(−)
Occult blood	(−)

Urine sedimentation	
Red blood cells, /high power field	< 1
White blood cells, /high power field	< 1

Urine chemistry	
Protein, g/g of creatinine	0.17

Complete blood cell counts	
White blood cells, /*μ*L	16,580
Neutrophil, %	90.8
Red blood cells, × 10^4^/*μ*L	388
Hemoglobin, g/dL	11.6
Platelets, × 10^4^/*μ*L	33.0

Serum chemistry	
Total protein, g/dL	6.6
Albumin, g/dL	3.8
Aspartate aminotransferase, IU/L	13
Alanine aminotransferase, IU/L	13
Lactate dehydrogenase, IU/L	176
Urea nitrogen, mg/dL	18.7
Creatinine, mg/dL	1.09
Total cholesterol, mg/dL	164
Triglyceride, mg/dL	95
Sodium, mEq/L	134
Potassium, mEq/L	4.5
Chloride, mEq/L	99
Calcium, mg/dL	9.6

Metabolic and endocrinological test	
Hemoglobin A1c, %	6.2

Serum immunological test	
C‐reactive protein, mg/dL	8.15
IgG4, mg/dL	16
Soluble interleukin‐2 receptor, U/mL	471.0

Coagulation test	
Prothrombin time, international normalized ratio	0.93
Activated partial thromboplastin time, sec	29.4
Fibrinogen, mg/dL	637
D‐dimer, *μ*g/mL	1.0

### 2.3. Clinical Course

The clinical course is shown in Figure 2. He was diagnosed with a suspected infected aortic aneurysm and started on antibiotic therapy. 2 weeks later, serum C‐reactive protein level decreased to 1.73 mg/dL; however, no bacteria were identified in blood cultures obtained before the initiation of antibiotic therapy. 6 weeks later, despite continued antibiotic therapy, serum C‐reactive protein levels increased again, and both the aneurysm and the surrounding tissue masses showed further expansion (Figure 1C). Positron emission tomography imaging demonstrated markedly increased accumulation of fluorine‐18 fluoro‐deoxyglucose at the margin of the abdominal aortic aneurysm, with a maximum standardized uptake value of 49.6 (Figure 3).

**Figure 2:**
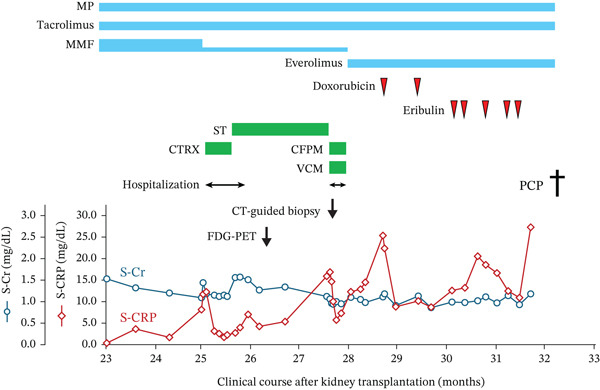
Clinical course. MP denotes methylprednisolone; MMF, mycophenolate mofetil; ST, sulfamethoxazole/trimethoprim; CTRX, ceftriaxone; CFPM, cefepime; VCM, vancomycin; S‐Cr, serum creatinine; S‐CRP, serum C‐reactive protein; CT, computed tomography; FDG‐PET, fluoro‐deoxyglucose‐positron emission tomography; PCP, Pneumocystis jirovecii pneumonia.

**Figure 3:**
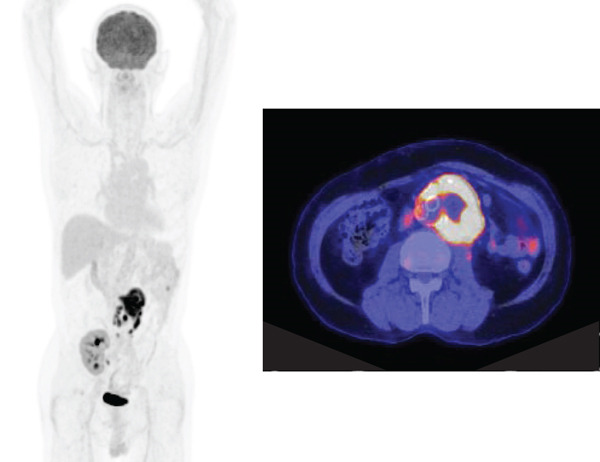
Fluoro‐deoxyglucose‐positron emission tomography findings 2 years and 2 months after the kidney transplantation. Positron emission tomography showed highly increased accumulation of fluorine‐18 fluoro‐deoxyglucose at the margin of the abdominal aortic aneurysm (standardized uptake value: 49.6).

### 2.4. Diagnosis of Spindle Cell Sarcoma

A computed tomography‐guided percutaneous needle biopsy was performed to obtain tissue from the tumor. Pathological findings demonstrated proliferation of spindle‐shaped neoplastic cells arranged in sheets and fascicles (Figure 4A). Scattered large pleomorphic cells with irregular and angulated nuclei were admixed, and mitotic figures were increased (Figure 4B). No definite vascular channel formation was identified. Based on the tumor location within the aortic wall and its high‐grade spindle cell morphology, angiosarcoma, intimal sarcoma, and leiomyosarcoma were considered the primary differential diagnoses.

**Figure 4:**
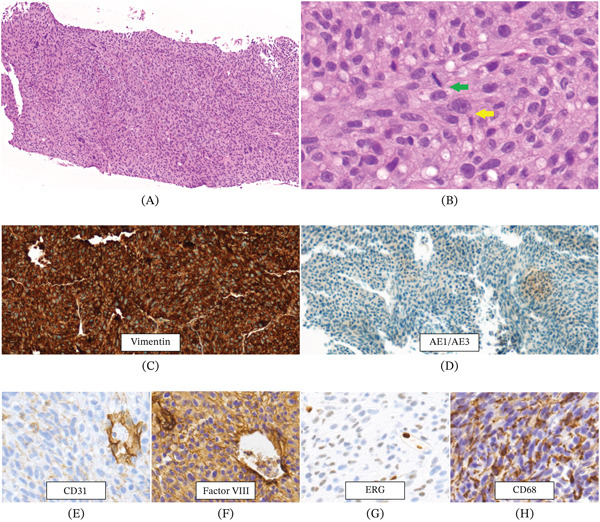
Pathological findings of the needle biopsy specimens. (A) Spindle‐shaped neoplastic cells arranged in sheets and fascicles (hematoxylin‐eosin stain; original magnification × 40). (B) Scattered large pleomorphic cells with distinct nucleoli (yellow arrow) and mitotic figures (green arrow) were admixed (hematoxylin‐eosin stain; original magnification × 400). (C) Spindle‐shaped neoplastic cells were positive for vimentin (immunoenzyme stain for vimentin; original magnification × 100). (D) Spindle‐shaped neoplastic cells were negative for cytokeratin AE1/AE3 (immunoenzyme stain for AE1/AE3; original magnification × 100). (E) Tumor cells were weakly positive for CD31, but the staining intensity was weaker than that of nontumor vascular endothelium, an endogenous positive control (immunoenzyme stain for CD31; original magnification × 400). (F) Tumor cells were weakly positive for factor VIII (immunoenzyme stain for factor VIII; original magnification × 400). (G) Tumor cells were weakly and focally positive for ERG in nuclei (immunoenzyme stain for ERG; original magnification × 400). (H) Numerous CD68‐positive cells interspersed among the tumor cells were present (immunoenzyme stain for CD68; original magnification × 400).

Immunohistochemically, the tumor cells were positive for vimentin, a mesenchymal marker (Figure 4C), and negative for cytokeratin AE1/AE3, an epithelial marker (Figure 4D), supporting a diagnosis of sarcoma. Tumor cells were weakly positive for CD31, but the staining intensity was weaker than that of nontumor vascular endothelium, an endogenous positive control (Figure 4E). The tumor cells were negative for CD34 and podoplanin, with only weak and focal positivity for factor VIII, and erythroblast transformation specific‐related gene (ERG) was observed (Figure 4F,G). Mouse double minute 2 (MDM2) and cyclin‐dependent kinase 4 (CDK4) were negative, and no MDM2 amplification was detected on genetic analysis, making intimal sarcoma unlikely. Smooth muscle markers, including smooth muscle actin (SMA), desmin, and calponin were also negative, excluding leiomyosarcoma. In addition, other spindle cell neoplasms were considered in the differential diagnosis. Peripheral nerve sheath tumors and melanoma were excluded by negative staining for S100 and SOX10. Synovial sarcoma was unlikely given the absence of SS18‐SSX expression, and solitary fibrous tumor was excluded by negative STAT6 staining. Numerous CD68‐positive and CD163‐positive cells were present (Figure 4H); however, these were interpreted as admixed histiocytes interspersed among the tumor cells rather than neoplastic components. As no definitive lineage‐specific markers were identified, and the focal expression of CD31 and factor VIII were insufficient to establish endothelial differentiation, a definitive diagnosis of angiosarcoma could not be made; therefore, the tumor was diagnosed as an undifferentiated spindle cell sarcoma of unknown lineage differentiation. Although scattered pleomorphic cells were observed, they were limited in number, and the overall histological pattern in the biopsy specimen was not entirely characteristic of undifferentiated pleomorphic sarcoma.

At the time of pathological diagnosis, the maximum diameter of the tumor was 105 mm, and it was classified as cT3 according to the American Joint Committee on Cancer/Union for International Cancer Control TMN staging system for soft tissue sarcomas. Based on available imaging, no regional lymph node or distant metastases were evident, and the patient was classified as cN0 and cM0.

### 2.5. Treatment Course and Clinical Outcome

The clinical course is summarized in Figure 2. Following the diagnosis of sarcoma, the immunosuppressive regimen was modified by switching mycophenolate mofetil to everolimus. Surgical resection was considered, but the tumor was growing rapidly, and extended resection of the tumor, including the stent graft, was deemed not feasible. Systemic chemotherapy with doxorubicin, followed by eribulin was initiated. However, the tumor continued to grow (Figure 1D), and he ultimately died due to Pneumocystis jirovecii pneumonia 7 months following the initial admission.

## 3. Discussion

### 3.1. EVAR Prior to Kidney Transplantation

A recent report stated that the frequency of aortoiliac aneurysms in patients with kidney transplant was 0.42% [8]. Furthermore, aortoiliac angiography prior to kidney transplant revealed abdominal aortic aneurysm requiring vascular reconstruction in 1.1% of candidates [9]. Therefore, careful evaluation for aortic aneurysms is recommended during pretransplant assessment. Even in patients with concomitant aortic aneurysms, EVAR does not preclude kidney transplantation, provided that vascular anastomosis for the renal graft is technically feasible [10].

Although reports remain limited, long‐term survival and graft function after EVAR in kidney transplant recipients appear comparable to those of nontransplant patients [3, 11]. Although EVAR in kidney transplant candidates may increase the risk of perioperative acute kidney injury, the 5‐year survival rate and long‐term graft function are comparable to those in nontransplant populations [12, 13].

In addition to EVAR, another treatment option for abdominal aortic aneurysms is open surgical repair; this may make it difficult to continue peritoneal dialysis. In our case, EVAR was performed for the abdominal aortic aneurysm, enabling the continuation of peritoneal dialysis and subsequent kidney transplantation. However, this can highlight the need for careful long‐term surveillance for late complications following EVAR.

### 3.2. Spindle Cell Sarcoma Following Kidney Transplantation

Except for Kaposi sarcoma, sarcomas following kidney transplantation are extremely rare [6]. In particular, angiosarcomas in kidney transplant recipients have characteristic sites of occurrence. Ahmed and Hamacher reported that five (38%) of 13 postkidney transplant patients developed angiosarcomas arising from arteriovenous fistula used for hemodialysis vascular access [14]. Conversely, in reported cases of angiosarcoma arising from arteriovenous fistula, 19 (86%) of the 22 cases occurred in patients who had undergone kidney transplantation [15]. Posttransplant immunosuppression and altered hemodynamic stress associated with arteriovenous fistulas or shunts are considered potential risk factors for the development of angiosarcoma [16]. Angiosarcomas following kidney transplantation have also been reported in the skin, liver, and kidney allograft [14, 17, 18]. To our knowledge, no previous reports have described spindle cell sarcoma arising with a native abdominal aortic aneurysm sac following EVAR, as observed in the present case.

Although the evidence regarding angiosarcoma following kidney transplantation is currently limited to case reports and case series, the vast majority of reported cases have resulted in death within a short period (1–15 months), indicating an extremely poor prognosis [14].

### 3.3. Spindle Cell Sarcoma Following EVAR

Primary aortic neoplasms are predominantly sarcomas, with the following histopathological subtypes reported: undifferentiated (40%), angiosarcomatous (37%), leiomyosarcoma (13%), and fibroblastic (7%) [4, 19]. Primary aortic sarcomas are extremely rare, but several reports describe aortic sarcomas following EVAR. Cases of sarcoma occurring at or around the aneurysm sac after EVAR include angiosarcoma, epithelioid angiosarcoma, and intimal sarcoma [20–24]. These often occur several years after EVAR and are frequently misdiagnosed as infection, inflammation, or recurrent aneurysm. The diagnosis of aortic sarcoma following EVAR is particularly challenging because no pathognomonic imaging or laboratory findings have been established.

The etiology of aortic sarcoma following EVAR remains uncertain. Nevertheless, circumstantial evidence suggests a potential association between biomedical implants and the development of angiosarcoma [25]. Animal experiments conducted long ago have implicated the exposure to plastic polymers can induce sarcomas [26], and previous reports have documented sarcoma cases arising around Dacron, a polyethylene terephthalate, grafts [27]. Chronic inflammatory responses to prosthetic materials can promote tissue remodeling and genetic mutations, potentially leading to malignant transformation [25]. The stent graft used in our case was primarily consisted of PTFE/FEP, fluorocarbon polymers, and nitinol, an alloy of nickel and titanium. There are fewer reported cases of sarcomas associated with PTFE compared to Dacron. The patterns of foreign body reactions may differ between PTFE and Dacron; whereas PTFE exhibits fibrotic reactions with encapsulation, Dacron may induce more severe inflammatory responses [28]. Although nickel has been recognized as carcinogenic with high‐concentration and long‐term occupational exposures [29], no carcinogenicity has been clinically reported from exposure to medical nickel‐titanium alloys. However, in vitro studies suggest that nickel‐titanium alloys cause interleukin‐1*β* secretion from monocytes, activating them and potentially promoting inflammatory responses in soft tissues [30]. In our patient, numerous CD68‐positive and CD163‐positive cells were present among the tumor cells. It has been shown that CD68‐positive and CD163‐positive macrophages are increased in angiosarcoma tissues, suggesting that M2‐polarized tumor‐associated macrophages may play a potential role in the progression of sarcomas [31].

This case was pathologically diagnosed as an undifferentiated spindle cell sarcoma of unknown direction of differentiation. MDM2 and CDK4, specific markers of intimal sarcoma, were negative, thus ruling out it. Similarly, CD34 and podoplanin were negative, but CD31, factor VIII, and ERG, endothelial cell markers, were weakly positive, suggesting that angiosarcoma remained a diagnostic consideration.

## 4. Conclusion

We report a case of a kidney transplant recipient who developed undifferentiated spindle cell sarcoma within an abdominal aortic aneurysm following EVAR. Although rare, sarcoma should be considered in the differential diagnosis, in addition to infection, endotension, or thrombosis, when transplant recipients with a history of EVAR present with aneurysm enlargement or inflammatory findings.

## Funding

No funding was received for this manuscript.

## Disclosure

All authors have reviewed and approved this manuscript using the 2013 CARE checklist.

## Ethics Statement

All procedures performed in this case were in accordance with the ethical standards of the 1964 Helsinki Declaration. Informed consent was obtained from the patient during his lifetime for the publication of this case report.

## Conflicts of Interest

The authors declare no conflicts of interest.

## Data Availability

Data sharing is not applicable to this article as no datasets were generated or analyzsed during the current study.
